# Recent advances in metal-organic frameworks for therapeutic applications in periodontitis

**DOI:** 10.3389/fmed.2025.1706849

**Published:** 2025-11-17

**Authors:** Jiaqi Chen, Yang Bai, Yude Ding, Linhong Wang, Wentao Zhang, Fan Yang

**Affiliations:** 1 Department of Stomatology, Center for Plastic and Reconstructive Surgery, Zhejiang Provincial People’s Hospital (Affiliated People’s Hospital), Hangzhou Medical College, Hangzhou, Zhejiang, China; 2 Zhejiang Provincial Clinical Research Center for Head and Neck Cancer, Hangzhou, China; 3 Zhejiang Key Laboratory of Precision Medicine Research on Head and Neck Cancer, Hangzhou, China; 4 School of Public Health, Zhejiang University School of Medicine, Hangzhou, Zhejiang, China

**Keywords:** metal-organic framework, periodontitis, antibacterial effect, anti-inflammation, tissue regeneration

## Abstract

Periodontitis is a highly prevalent chronic inflammatory disease that leads to the destruction of periodontal tissues and has systemic health implications. Current treatments, such as mechanical debridement and antibiotics, are often inadequate due to poor biofilm penetration, drug resistance, and limited regenerative capacity. Metal-organic frameworks (MOFs) have recently emerged as a transformative platform to address these limitations and surpass traditional nanomaterials (e.g., liposomes, dendrimers). Their tunable porosity, multifunctional composition, and responsive degradation enable antibacterial, anti-inflammatory, and pro-regenerative actions. This review highlights the mechanisms by which MOFs combat periodontal pathogens, resolve inflammation, and promote tissue regeneration, outperforming conventional materials. We also discuss combinatorial strategies, such as photodynamic and immunomodulatory therapies, that enhance treatment efficacy. Despite promising preclinical results, challenges in biocompatibility, biodegradation, and clinical translation remain. Future efforts should focus on developing intelligent, multifunctional MOF platforms capable of microenvironment-responsive therapy to achieve complete periodontal restoration.

## 1 Introduction

Periodontitis is a severe gum infection caused by an imbalance of oral bacteria. It is characterized by progressive connective tissue degradation, alveolar bone resorption, and eventual tooth loss (1). Affecting approximately 10–15% of the global population, it ranks as the sixth most common disease worldwide. Notably, severe periodontitis exhibits systemic associations with cardiovascular diseases, type 2 diabetes mellitus, rheumatoid arthritis, inflammatory bowel disease, Alzheimer’s disease, non-alcoholic fatty liver disease and certain malignancies (2–4). Given its high prevalence and multisystemic implications, periodontitis represents a significant public health challenge with profound socioeconomic consequences.

Treating periodontitis requires a strategy that combats infection, controls inflammation, and regenerates lost tissue. Standard treatments include deep cleaning (scaling and root planning) and applying antibiotics directly into gum pockets. However, these methods have significant drawbacks. Surgery is costly and invasive, antibiotics are becoming less effective due to resistance, and these approaches do not effectively promote the regrowth of healthy tissue. Moreover, antibiotic regimens—including next-generation agents—face unavoidable limitations such as the emergence of microbial drug resistance and restricted antimicrobial spectrum. These challenges underscore urgent need for non-surgical, antibiotic-free therapeutic modalities capable of rapidly eliminating oral biofilms while promoting accelerated periodontal tissue regeneration. The treatment strategies and mechanisms of action for periodontitis are summarized in Table 1.

**TABLE 1:** Mechanism strategy of periodontitis.

Therapy	Mechanism	Advantage
Scaling and root planning	Biofilm control	Eliminating pathogenic factors and control inflammation
Mouthrinses containing chlorhexidine et al.	Biofilm control	Assisting in plaque removal
Antimicrobials/ probiotics/host modulation	Antibacterial effect	Reducing side effects of mechanical debridement, including gingival recession
Periodontal surgery	Completely clearing infection, recovering periodontal tissue and function	Consolidating the effect of periodontal treatment and achieve periodontal tissue regeneration
supportive periodontal therapy	Monitoring periodontal condition	Maintaining the long-term efficacy

**FIGURE 1:**
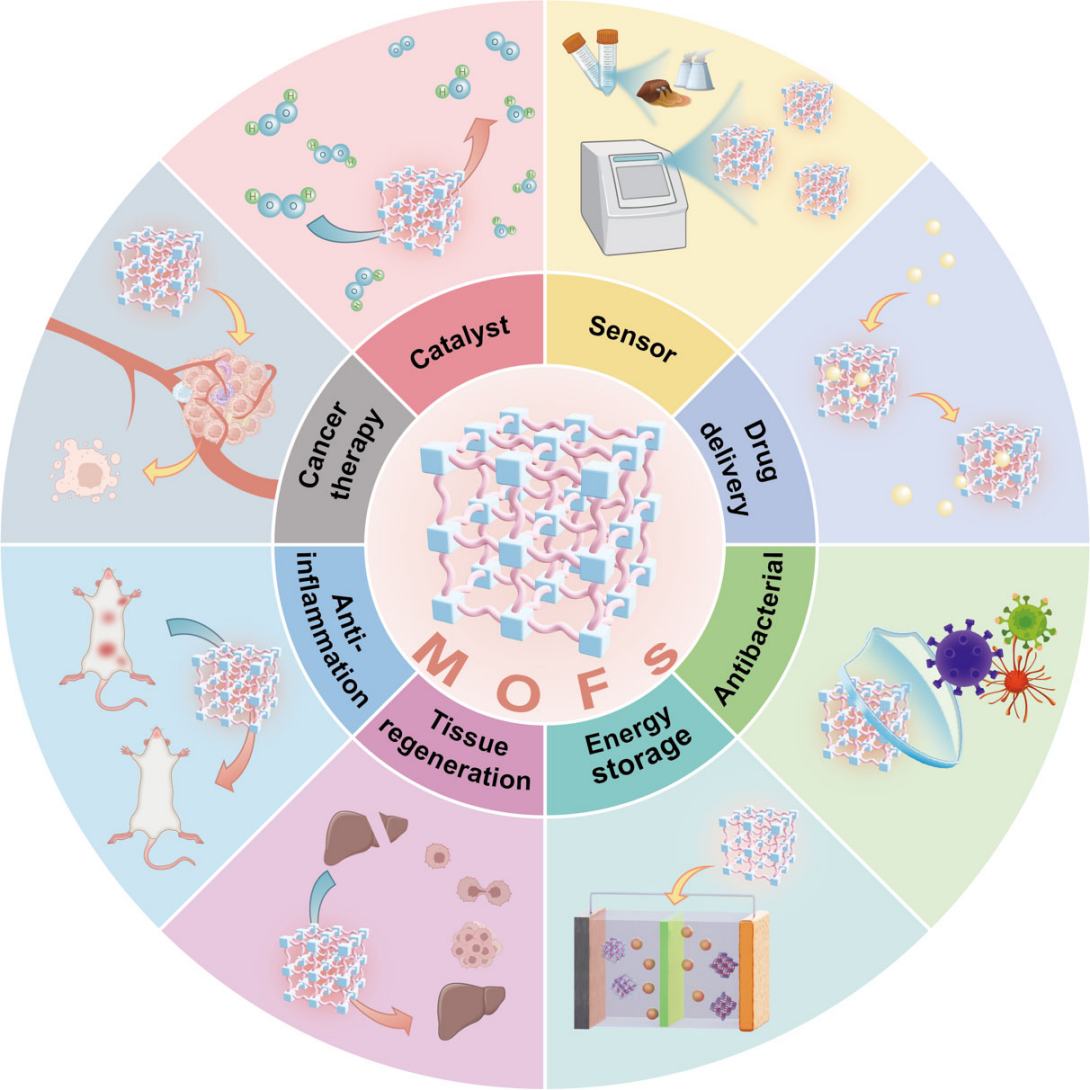
Schematic diagram of multifaceted applications of MOFs. Schematic illustration of the diverse frontier applications of MOFs. As advanced crystalline porous materials constructed via coordination between metal ions/clusters and organic bridging ligands, MOFs exhibit prominent utility in catalysis, sensing, drug delivery, antibacterial activity, energy storage, tissue regeneration, anti-inflammation, and cancer therapy, highlighting their structural versatility and multifunctional potential.

**FIGURE 2:**
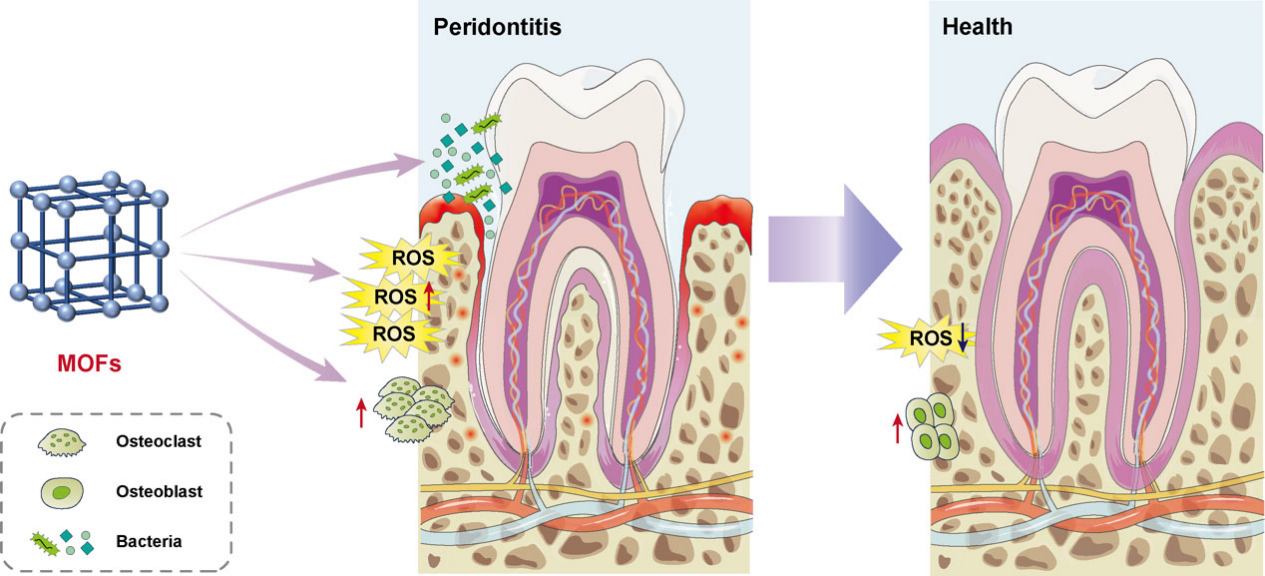
Schematic diagram of the mechanism of MOFs in treating periodontitis. This schematic diagram illustrates the therapeutic mechanism of MOFs in periodontitis. The MOF crystal structure (left) regulates the periodontal tissue microenvironment through three synergistic pathways: antibacterial action, oxidative stress modulation, and bone metabolism regulation. Ultimately, MOFs restore diseased periodontal tissue to a healthy state.

Metal-organic frameworks (MOFs) are highly porous materials made from metal ions and organic linkers, which have garnered extensive attention across multiple disciplinary fields (5) (Figure 1). Their application scope spans a broad range of critical research and industrial domains, encompassing heterogeneous catalysis, environmental sensing, energy storage, and biomedical engineering (including targeted drug delivery, antibacterial treatment, anti-inflammatory therapy, and cancer therapy). Owing to the exceptional structural flexibility and functional adaptability, MOFs emerged as promising candidates in periodontal therapeutics (6–10). By virtue of their bioactive constituents, MOFs exhibit remarkable potential for anti-inflammatory applications and controlled drug delivery. Specifically, MOFs exert bactericidal effects through the release of metal ions, while modified MOFs possess immunomodulatory and osteogenic properties, playing critical roles in periodontitis treatment (8, 11). This review systematically examines the pathogenesis of periodontitis while elucidating recent advances in MOF-based therapeutic strategies, with particular emphasis on their molecular mechanisms of action (Figure 2). This review critically evaluates the current applications of MOFs in periodontal interventions, with a particular focus on distinguishing foundational studies that established key therapeutic functions, such as antimicrobial ion release, from transformative platforms that achieve systematic integration of these functions into intelligent, multifunctional systems for coordinated and modulated therapy. The potential benefits of MOFs in microbial control, inflammation resolution, and tissue regeneration are delineated, while limitations including long-term biocompatibility concerns, biodegradation kinetics, and scale-up challenges are discussed to inform future translational research directions.

## 2 The therapeutic roles of MOFs in periodontitis

The management of periodontitis necessitates a multimodal therapeutic strategy that synergistically combines antibacterial, anti-inflammatory, and regenerative approaches, targeting both pathogenic mechanisms and tissue repair processes. In the field of periodontitis treatment, ideal healing is no longer confined to the mere alleviation of clinical inflammatory symptoms achieved through traditional mechanical debridement. Instead, it refers to a more profound biological process-the integration of infection control, restoration of immune homeostasis, and functional regeneration of periodontal tissues. The core objectives encompass the complete eradication of pathogenic bacteria, precise modulation of the host immune response, reconstruction of the three-dimensional architecture of periodontal supporting tissues (alveolar bone, periodontal ligament, and cementum), and ultimately, the reestablishment of stable periodontal attachment to achieve long-term tissue homeostasis. Conventional periodontitis treatments like mechanical debridement, local antibiotics, and flap surgery, though capable of mitigating acute inflammation, are inherently limited by their passive approach. These include an inability to actively regulate the host immune response, difficulty in thoroughly eliminating deep-seated pathogens, risks associated with antibiotic burst release and resistance, and insufficient guidance for periodontal ligament stem cell-mediated regeneration. Crucially, they lack the capacity to promote the functional regeneration of structures like alveolar bone and periodontal ligament, often resulting in incomplete repair and high recurrence rates.

The pursuit of a multimodal therapeutic strategy for periodontitis has driven the innovation of MOF-based nanomaterials, which are uniquely positioned to concurrently address bacterial infection, rampant inflammation, and tissue destruction. Representative examples of these platforms are systematically compared in Table 2, providing a high-level summary of their compositions, mechanisms, and primary functions. The subsequent sections are dedicated to unpacking the evidence for each of these pivotal functions—antibacterial, anti-inflammatory, and regenerative—which are often intricately linked and exhibited in concert by a single MOF formulation, as illustrated in Figure 2 and Table 3. Recent advances have established MOFs as a transformative platform for periodontal therapy, effectively overcoming key clinical limitations through multifunctional capabilities (9, 12–14). In contrast to systemic antibiotics—whose indiscriminate use exacerbates antimicrobial resistance (15), MOFs offer a revolutionary active-regeneration strategy for periodontal healing, leveraging their precisely designable structures and multifunctional integration. Their structural precision enables multiple mechanisms. They function as intelligent drug delivery systems for sustained therapeutic release. They actively remodel the inflammatory milieu into a pro-regenerative niche by modulating signaling pathways like Nuclear factor erythroid 2-related factor 2/Nuclear factor kappa-light-chain-enhancer of activated B cells (Nrf2/NF-κB) and Wnt (16, 17). Concurrently, they serve as biomimetic scaffolds that support cell adhesion and differentiation while delivering growth factors (18, 19). Finally, they undergo gradual biodegradation after fulfilling their therapeutic role, ensuring a favorable safety profile (20, 21). Whereas most available biomaterials for periodontitis possess singular functions (22, 23), MOFs’ engineerable structures allow for customized multipronged therapy through surface modification, functional fusion, and carrier integration (e.g., hydrogels, scaffolds) (24). To this end, the following sections detail the antibacterial, anti-inflammatory, and regenerative capabilities of MOFs. These interrelated functions are systematically compared in Tables 2, 3 and summarized in Figure 2. Through these coordinated actions, MOF-based platforms guide synchronized regeneration of periodontal soft and hard tissues, ultimately achieving the paramount goal of “periodontal reattachment”.

**TABLE 2:** Representative MOF-based platforms for periodontitis therapy.

MOFs	Components	Target	Mechanism	Key functions
CuTCPP-Fe_2_O_3_	Cu^2+^, Fe^3+^, TCPP ligand	ROS, bacterial cells	PDT, metal ion release	Broad-spectrum antibacterial, anti-inflammatory, angiogenesis promotion
DZIF@PGel	ZIF-8, DEX, GelMA, PPEMA	Zn^2+^, inflammatory pathways	Fenton-like reaction, sustained drug release	Antibacterial, anti-inflammatory, osteogenesis
MPB-BA	MPB, BA	ROS, macrophages	PTT, antioxidant drug release, immunomodulation	Antibacterial, antioxidant, immunomodulation (M1 to M2 polarization)
GelMA-Z	GelMA, ZIF-8	Zn^2+^, bacteria, osteoblasts	Fenton-like Reaction, Sustained Zn^2+^ Release, Osteogenic Induction	Antibacterial, anti-inflammatory, alveolar bone regeneration
I_2_@COF-HEC	COF, I2, HEC	I2, bacteria	Sustained I2 release, direct bactericidal action	Inhibits alveolar bone resorption, reduces periodontal pocket depth

**TABLE 3:** Mechanisms of action of MOFs in periodontitis therapy.

Mechanism	Key modes of action	Primary effects
Ion release	Sustained release of bioactive ions (e.g., Zn^2+^, Cu^2+^, Mg^2+^, Ag^+^); Controlled degradation of the MOF structure.	Disrupts bacterial cell membranes and inhibits enzyme activity (antibacterial); Promotes osteogenic differentiation of BMSCs (regenerative); Modulates immune cell response (anti-inflammatory).
ROS regulation	ROS Generation: Acts as a nanozyme in Fenton/Fenton-like reactions to produce OH from H_2_O_2_; ROS Scavenging: Mimics antioxidant enzymes (SOD, CAT) to decompose excess ROS.	Induces oxidative damage to pathogens (antibacterial); Alleviates oxidative stress in host tissues, reducing inflammation and promoting a regenerative microenvironment.
Macrophage polarization	Reprograms macrophages from the pro-inflammatory M1 phenotype to the anti-inflammatory M2 phenotype; Often mediated by released metal ions (e.g., Mg^2+^) or loaded anti-inflammatory drugs (e.g., DEX).	Decreases pro-inflammatory cytokines (e.g., IL-1β, TNF-α, iNOS); Increases anti-inflammatory cytokines (e.g., IL-10, TGF-β); Creates an immunomodulatory milieu conducive to healing.
Angiogenesis	Ions like Co^2+^ and Ni^2+^ mimic hypoxia, upregulating HIF-1α and VEGF expression; MOFs act as carriers for pro-angiogenic factors (e.g., VEGF, exosomes).	Stimulates the formation of new blood vessels; Provides essential oxygen, nutrients, and growth factors for successful bone and tissue regeneration.

### 2.1 Antimicrobial effect

The development of periodontitis starts with dental plaque, a sticky film of bacteria that contains harmful species, such as *Porphyromonas gingivalis* (*P. gingivalis*), *Fusobacterium nucleatum* (*F. nucleatum*), and *Staphylococcus aureus* (*S. aureus*) (25–27). It is hard to eliminate these bacteria because they form protective communities called biofilms and have natural ways to resist antibiotics. Leveraging the unique antibacterial properties of MOFs, researchers have developed MOF-based therapeutic strategies for periodontitis and made notable progress in this field. This section primarily explores the antibacterial mechanisms and research advancements of MOF-based materials in various antibacterial applications, with a particular focus on periodontitis.

MOFs are effective antibacterial agents for three main reasons: (1) The metal parts (like silver or zinc) can kill bacteria (28–30). (2) Their tiny pores can trap and carry antibiotic drugs. (3) Some MOFs can be activated by light to produce bacteria-killing substances, thereby ablating bacterial cells through reactive oxygen species (ROS) generation.

In addition to copper and iron ions, which have been applied in the antibacterial therapy of periodontitis, Yang et al. also revealed that Mg^2+^ and Zn^2+^ exhibit remarkable antibacterial activities (31). Zn^2+^ exerted direct antibacterial effects by disrupting bacterial cell membrane integrity and inhibiting bacterial enzyme activity. Moreover, it synergized with Mg^2+^ to mediate pyroptosis, suppress lipopolysaccharide (LPS)-induced inflammatory responses, and create an environment unfavorable for bacterial colonization and infection. This dual mechanism reduced the opportunity for bacteria to activate host cell pyroptosis via LPS, thereby decreasing the persistence of bacterial infection.

In the study by Li et al., the authors highlight the capability of two-dimensional metal–organic frameworks to enable sustained release of bioactive ions at the infection site, thereby maintaining effective local concentrations over prolonged periods and reducing dosing frequency (31). This is quantitatively demonstrated by the ion release behavior of the CuTCPP-Fe2O3 nanocomposite: the cumulative Cu^2+^ release in PBS reached 0.485 ± 0.045 ppm within 24 h and increased gradually to 2.037 ± 0.075 ppm over 28 days, while Fe^3+^ release reached 0.145 ± 0.018 ppm at 24 h and 0.499 ± 0.015 ppm after 28 days, with no burst release observed. Such sustained ion release, corroborated by transmission electron microscopy (TEM) morphological analysis, underscored the structural stability and long-term degradability of the MOF-based system under physiological conditions. Beyond copper and iron ions, which contributed to antibacterial and pro-angiogenic effects, other metal ions such as Mg^2+^ and Zn^2+^ have also been shown to exert remarkable antibacterial activities. As reported by Yang et al., Zn^2+^ disrupted bacterial membrane integrity and inhibited enzymatic activity, while synergizing with Mg^2+^ to induce pyroptosis and suppress LPS-induced inflammatory responses (13). This combined action limited bacterial colonization and mitigates infection persistence. The integration of multiple bioactive metal ions into a stable MOF platform not only extends local antimicrobial and anti-inflammatory action but also minimizes the need for frequent administration, offering a promising strategy for long-term management of periodontal diseases. While these studies solidly established the foundational “toolbox” of bioactive metal ions and their mechanisms, they primarily represent the first step in leveraging MOFs for periodontitis. Their therapeutic outcome is often contingent on the passive release of ions, which lacks the spatiotemporal control and multifunctional integration seen in more advanced platforms.

Besides releasing ions, MOFs can also kill bacteria by generating heat when exposed to light, or by delivering loaded antibiotic drugs. Tian et al. demonstrated that mesoporous prussian blue (MPB) loaded with baicalein (BA), an antibacterial compound, could be fabricated into a versatile MOF-based nanoplatform (MPB-BA) (16). This combined effect of thermal ablation and concurrently enhanced local drug delivery results in a more comprehensive bactericidal outcome against periodontopathogens. Under near-infrared (NIR) light irradiation, MPB-BA could reach a temperature of 88.8°C. Temperatures exceeding 50°C typically disrupted bacterial cell membranes and denature proteins, thereby inhibiting or killing bacteria. BA itself possessed antibacterial properties; MPB delivered BA to the inflamed site, where NIR light triggered BA release to synergize with MPB’s photothermal effect for combined bacterial growth inhibition. The antibacterial activity of MPB-BA under NIR light was validated via plate counting assays against P. gingivalis and F. nucleatum, o-nitrophenyl-β-D-galactopyranoside membrane permeability assays, and scanning electron microscopy observations.

Photothermal therapy (PTT) and photodynamic therapy (PDT) have emerged as clinically viable strategies for antibacterial applications, leveraging the ability of photosensitizers to generate ROS that rapidly eradicate pathogens (32). Porphyrin-based MOFs further optimize this process by enhancing light-harvesting efficiency and ROS generation (33, 34). A notable example is the CuTCPP-Fe2O3, which exerted a dual-mode antibacterial action by simultaneously generating bactericidal ROS through photodynamic action and releasing bioactive Fe^3+^/Cu^2+^ ions. This combined chemical and ionic assault inflicts comprehensive damage on bacterial cells, demonstrating broad-spectrum activity (31). In a more targeted approach, the UBI29-41-functionalized MOF platform (ICG@Uio-66-UBI) achieved precision PDT by selectively binding to bacterial surfaces via electrostatic interactions (35). Under 808 nm NIR irradiation, it induced localized ROS production, resulting in an approximate 2 log reduction in CFUs against periodontal biofilms and a significant increase in nucleic acid leakage (OD260), confirming membrane disruption. Furthermore, the platform extended its anti-virulence action by downregulating the LuxS gene to 21.6% of control levels and inhibiting AI-2 quorum sensing signaling. These results underscored the capability of MOF-based PDT platforms to integrate targeted bacterial elimination with virulence suppression, offering a sophisticated alternative to traditional antibiotics for treating periodontitis.

Collectively, these studies underscore the multifaceted antibacterial potential of MOFs against periodontitis-associated pathogens. By leveraging intrinsic properties (metal ion release) or engineered functionalities (PTT conversion, PDT activation, drug encapsulation), MOFs overcome biofilm resistance and achieve potent bactericidal effects. This evolving paradigm offers a promising alternative to conventional antibiotics, warranting further clinical exploration despite translational challenges that must be systematically addressed to realize its full clinical potential.

### 2.2 Immunomodulatory and anti-inflammatory effect

In periodontitis, the immune system overreacts, producing too many inflammatory signals, such as interleukin-6 (IL-6), interleukin-1beta (IL-1β), and tumor necrosis factor-alpha (TNF-α). The elevated ROS collectively drive osteoclast activation and alveolar bone resorption. A key problem is a change in macrophage type (from the healing M2 type to the damaging M1 type). This change, along with oxidative stress, creates a vicious cycle that makes the inflammation and tissue damage worse (36). In advanced stages, overactivated macrophages and neutrophils release sustained waves of pro-inflammatory mediators, leading to bone destruction, necrosis, and fibrous tissue proliferation (37). These observations underscore the therapeutic rationale for simultaneously targeting cytokines and ROS to mitigate osteoclast-mediated bone loss and oxidative stress.

Normally, bone tissue has low levels of ROS for cell communication and defense. However, during inflammation, ROS levels rise sharply and overwhelm the body’s natural antioxidant defenses, including superoxide dismutase (SOD) and catalase (CAT). Excess ROS suppress osteogenic differentiation by downregulating key transcription factors such as runt-related transcription factor 2 (Runx2) and Osterix, while concurrently promoting osteoclastogenesis via the receptor activator of nuclear factor kappa-B ligand (RANKL)/receptor activator of nuclear factor kappa-B (RANK)/NF-κB axis (38, 39). This redox imbalance thus aggravates inflammatory damage and impairs the regeneration of periodontal support tissues. Therefore, beyond antimicrobial strategies, effective periodontitis therapy must incorporate interventions that alleviate oxidative stress and modulate immune overactivation.

**FIGURE 3:**
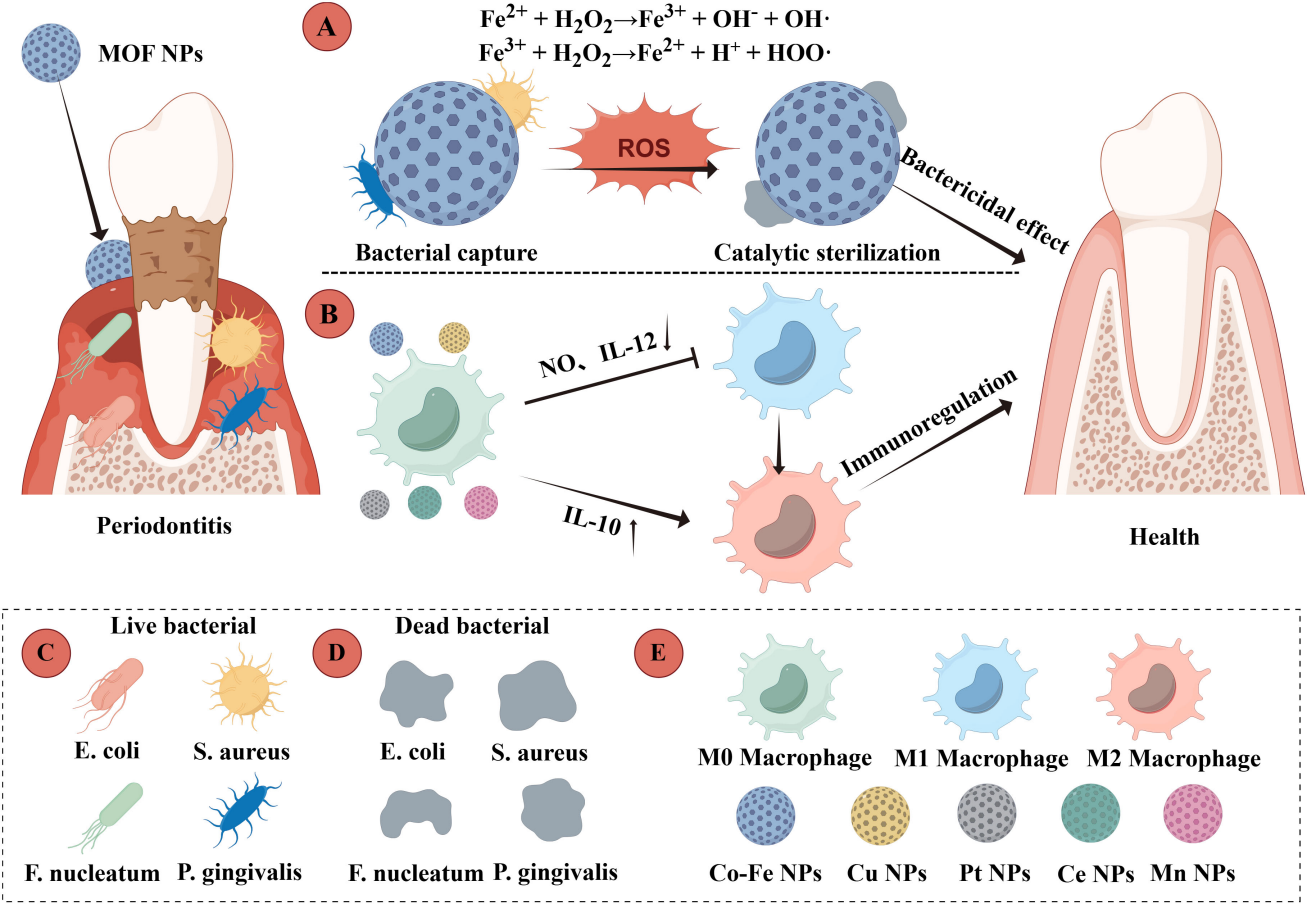
MOF-mediated Fenton reaction for periodontitis treatment: bactericidal and immunomodulatory mechanisms. **(A)** MOF nanoparticles catalyze the generation of bactericidal reactive oxygen species (ROS) through Fenton reactions. **(B)** Immunomodulatory cascade during inflammatory resolution. **(C)** Representative live periodontopathogenic bacteria. **(D)** Corresponding bacteria after MOF-mediated sterilization. **(E)** Phenotypes of macrophages involved in the immune response. The figure was created with Figdraw (www.figdraw.com).

MOFs have recently emerged as versatile nanoplatforms not only for antibacterial applications but also for immunomodulation. By cleaning up the excess ROS, some MOFs can break the cycle of inflammation and oxidative stress, helping to restore a balanced immune state (40). Moreover, MOFs can serve as targeted delivery systems for anti-inflammatory agents, offering a promising approach to mitigate bone tissue inflammation and facilitate periodontal recovery (Figure 3). As illustrated in Figure 3, this process involves a dual mechanism: MOF nanoparticles catalyze Fenton reactions to generate bactericidal ROS, while concurrently initiating an immunomodulatory cascade that promotes the resolution of inflammation by modulating macrophage polarization.

HMUiO-66-NH_2_ nanoparticles represent a marked advance in periodontitis therapy, embodying a multifunctional design that provides anti-inflammatory and immunomodulatory effects unattainable by conventional single-target approaches (41). The anti-inflammatory effect was mechanistically linked to their potent ROS-scavenging capability. By effectively decomposing H_2_O_2_ via a peroxidase-mimetic non-Fenton pathway, these nanoparticles alleviated the oxidative stress that drives NF-κB signaling and pro-inflammatory cytokine production. *In vivo* evidence directly supported this: in a ligature-induced periodontitis mouse model, local injection of HMUiO-66-NH_2_ significantly reduced gingival ROS levels, as quantified by fluorescence imaging, and subsequently attenuated inflammatory cell infiltration in periodontal tissues, as confirmed by hematoxylin and eosin (H&E) staining. Furthermore, immunomodulation was evidenced by the suppression of osteoclastogenesis, a key inflammatory bone resorption process. Tartrate-resistant acid phosphatase (TRAP) staining revealed a substantial decrease in the number of TRAP-positive osteoclasts in the high-dose HMUiO-66-NH_2_ treatment group compared to the saline-treated periodontitis control. Unlike conventional antioxidants that are incapable of promoting bone formation, HMUiO-66-NH_2_ exhibited a bifunctional mechanism: it neutralized the inflammatory trigger ROS while suppressing osteoclast activity. This dual capacity enabled a comprehensive therapeutic strategy, simultaneously resolving oxidative stress and stimulating bone regeneration through the upregulation of osteogenic genes and pathways [Wnt, transforming growth factor-beta (TGF-β)]. Consequently, it targeted both the cause and consequence of inflammatory bone loss, outperforming single-mechanism therapies.

Recent studies have shown that some nanomaterials containing dexamethasone, such as liposomes and hydrogels, exhibited dual anti-inflammatory and antibacterial functions (42, 43). Li et al. demonstrated that dexamethasone-loaded zeolitic imidazolate framework-8 nanocomposite hydrogels not only possessed antibacterial activity but also reduced ROS production via antioxidant effects of sustained-release Zn^2+^, indirectly inhibiting ROS-mediated inflammatory signaling pathways (8). The loaded dexamethasone (DEX) itself suppressed the activation of NF-κB signaling pathways, reducing transcription and release of inflammatory cytokines, decreasing infiltration of immune cells (e.g., macrophages, lymphocytes) at inflammatory sites, and promoting Runx2 expression in bone marrow mesenchymal stem cells (BMSCs) to counteract inflammation-induced inhibition of osteogenic differentiation. By reducing osteoclast activity, it alleviated inflammation-induced bone resorption, thereby achieving multi-dimensional regulation of the inflammatory microenvironment in periodontitis.

During periodontitis treatment, the dynamic complexity of the oral environment including salivary flow, chemical composition, and temperature fluctuations may cause rapid loss of MOF-based drugs (44). The irregular anatomy of periodontal pockets further adds therapeutic challenges, prompting researchers to combine MOFs with other materials for cooperative therapy (45). Luo et al. developed an intelligent responsive hydrogel-MOF system (CSBDX@MOF), where Mg-gallate (Mg-GA)-loaded MOF was incorporated into a hydrogel (14). This system modulated immune microenvironment by regulating macrophage polarization and inflammatory cytokines, alleviating periodontal inflammation and promoting tissue/bone regeneration. The hydrogel’s injectability and self-healing properties ensured sustained MOF release in periodontal pockets, synergistically inhibiting pathogen colonization and inflammatory stimulation. On-demand Mg-GA release enhanced local antibacterial/anti-inflammatory effects. Mechanistically, MOF-derived Mg^2+^ promoted macrophage polarization from pro-inflammatory M1 (iNOS +) to anti-inflammatory M2 (CD206 +). It activated RUNX2 to induce osteoblast differentiation, while inhibiting RANKL expression; promoted vascular endothelial growth factor (VEGF)/cluster of differentiation 31 (CD31) to support bone regeneration. Loaded gallate inhibited NF-κB pathway, reduced M1-secreted IL-6/cyclooxygenase-2 (COX-2), decreased intracellular ROS, and reconstructed immune microenvironment. Collectively, CSBDX@MOF optimized immune microenvironment, facilitating periodontal tissue repair and bone regeneration. The composite hydrogel SFD/CS/ZIF-8@QCT prepared by modifying ZIF-8 also exhibited effects through similar mechanisms in periodontitis treatment (12). It drove macrophage reprograming from M1 to M2 phenotype, effectively regulating the immune microenvironment while promoting the expression of osteogenic (OPN) and angiogenic (CD31) markers, alleviating local inflammation, and facilitating alveolar bone regeneration.

The poly(lactic-co-glycolic acid) (PLGA)/Exo-Mg-GA MOF composite scaffold demonstrated potent anti-inflammatory and immunomodulatory capabilities for periodontitis therapy (46). The Mg-GA MOF enabled sustained co-release of gallic acid (GA) and Mg^2+^ ions, which synergistically mitigated inflammation through dual mechanisms. GA directly suppressed pro-inflammatory mediators (iNOS, COX-2) via NF-κB inhibition while scavenging ROS to alleviate oxidative stress. Concurrently, Mg^2+^ ions modulated calcium signaling to further dampen inflammatory cascades. This combined action reduced osteoclast activity and promoted macrophage polarization toward the anti-inflammatory M2 phenotype, characterized by decreased IL-1β/interleukin-17 (IL-17) and elevated interleukin-10 (IL-10) expression through suppressed NF-κB/mitogen-activated protein kinase (MAPK) signaling. *In vivo* validation confirmed diminished inflammatory infiltration and reduced matrix metalloproteinase-9 (MMP9)/TNF-α levels, establishing an immunoregulatory microenvironment conducive to periodontal regeneration.

In addition to combining with various materials to optimize MOF properties, the photodynamic and photothermal effects of MOFs-beyond the antibacterial functions mentioned above-also exhibited anti-inflammatory and immunomodulatory activities. CuTCPP-Fe_2_O_3_ released ROS via photodynamic action and ions, which inhibited the NF-κB signaling pathway, significantly downregulating the expression of pro-inflammatory factors (iNOS, COX-2). Meanwhile, Cu^2+^ promoted collagen deposition and VEGF secretion, improving the local microenvironment. *In vivo* experiments confirmed that this MOF system reduced inflammatory cell infiltration in a rat periodontitis model, promoted angiogenesis by upregulating VEGF and CD31 expression, shortened the distance between the alveolar bone crest and cementoenamel junction (CEJ), and accelerated periodontal tissue repair, outperforming clinical antibiotic therapy. This photodynamic ion therapy provided an antibiotic-free strategy for periodontitis by dual actions of pathogen clearance and immune microenvironment regulation (31). The MPB-BA nanoplatform, beyond its antibacterial effects described earlier, achieved anti-inflammatory and immunomodulatory effects in periodontitis through multi-dimensional mechanisms (16). Under NIR light, the platform synergized MPB’s photothermal properties with BA’s pharmacological activity: it scavenged intracellular ROS, upregulated antioxidant genes (SOD-1, CAT) to block oxidative stress-induced inflammatory cascades, and regulated macrophage polarization from M1 to M2 phenotype, reducing pro-inflammatory factors (IL-1β, TNF-α, iNOS) while increasing anti-inflammatory factors (TGF-β, IL-10). In a rat model, the platform shortened alveolar bone resorption distance (CEJ-ABC) by reducing inflammatory cell infiltration and promoting VEGF expression, outperforming minocycline hydrochloride with good safety. It offered a nanotherapeutic strategy integrating photothermal antibacterial and immunomodulatory functions for periodontitis.

Aside from anti-inflammatory medications, natural signaling molecules are also employed to modulate the inflammatory microenvironment. Zheng et al. synthesized Zn/Mg-MOF with IL-4 encapsulation to control the inflammatory response in bone regeneration (47). The results showed that IL-4 was a prototypical anti-inflammatory cytokine that hindered the development of Th1 cells and decreased the release of pro-inflammatory cytokines via activating the Janus kinase (JAK)/signal transducer and activator of transcription (STAT) pathway. *In vitro* anti-inflammatory experiments demonstrated that it increased the production of CD206 and decreased the production of iNOS in RAW 264.7 macrophages, suggesting a decrease in inflammation.

### 2.3 Tissue regeneration

Periodontopathic bacteria not only induce inflammation but also inhibit fibroblast proliferation and activate osteoclast activity. Thus, the ultimate goal of periodontitis therapy is the functional regeneration of lost tissues. Regulation of bone tissue inflammation and defect repair can be achieved through four key mechanisms: (1) inducing osteogenic differentiation of BMSCs; (2) modulating inflammation and oxidative stress in bone tissue; (3) protecting periodontal tissues from infection; and (4) supporting adequate blood supply by promoting bone angiogenesis (48). Current research is exploring therapeutic strategies involving metal ions, signaling molecules, and pharmaceuticals. Emerging antibacterial nanomaterials such as MOFs can not only release metal ions but also deliver drugs and transmit signaling molecules to regulate the periodontitis microenvironment, significantly overcoming the limitations of conventional approaches (e.g., poor drug release control and susceptibility to inactivation/degradation) (49). Various MOFs have been applied to functionalize biomaterials for applications in osteoporosis treatment, bone regeneration, and bone defect repair.

A variety of bone-induced metal ions, such as Mg^2+^, Zn^2+^, and Ca^2+^, have been previously demonstrated to play critical roles in the activation of BMSCs and osteoblasts (50). For example, zinc-based MOFs (Zn MOFs), such as ZIF-8, have shown great potential to stimulate the osteogenic signaling pathway in BMSCs, thereby promoting osteogenic differentiation (19, 51). In a previous study, a mixed bilayer electrospun membrane that combined polycaprolactone and type I collagen was loaded with ZIF-8 nanoparticles to promote bone regeneration (52). The bone induction effect of ZIF-8 was evaluated. The real-time PCR results showed that the expression of osteogenic-related genes such as Runx2, osteocalcin (OCN), and alkaline phosphatase (ALP) was significantly upregulated in BMSCs. Another research revealed that ZIF-8 nanoparticles release Zn^2+^ to activate the extracellular signal-regulated kinase 1 (ERK1) and extracellular signal-regulated kinase 2 (ERK2) proteins in BMSCs, thereby initiating the downstream MAPK pathway and upregulating the expression of osteogenic-related genes (53).

In addition to Zn MOFs, magnesium-based MOFs (Mg MOFs) also demonstrate the potential to promote osteogenesis. Kang et al. mixed Mg MOF with PLGA to prepare PLGA/MOF electrospun membranes, which were then modified with exosomes extracted from adipose stem cells. It suggested that Mg^2+^ released from Mg MOFs activated the Notch and PI3K/AKT pathways in osteoblasts, promoting osteogenic differentiation of BMSCs (46).

ZIF-8-modified gelatin methacryloyl (GelMA) hydrogel (GelMA-Z) exhibited therapeutic effects on periodontitis through multiple mechanisms, with particularly notable efficacy in promoting tissue regeneration (8). The composite hydrogel remained liquid at body temperature, exhibiting excellent fluidity to allow injectable filling of periodontal pockets; upon UV irradiation, it rapidly crosslinked into a gel state for stable retention. Its porous structure facilitated cell migration and nutrient transport, with favorable cytocompatibility. Sustained release of Zn^2+^ from GelMA-Z not only downregulated inflammation-related gene expression via NF-κB pathway inhibition and modulated COX2 expression to alleviate inflammation but also promoted the expression of osteogenic differentiation-related genes and proteins. Concurrently, it inhibited bacterial colonization, providing continuous support for tissue regeneration. Through a tripartite mechanism simultaneously inhibiting bacterial colonization, dampening inflammation via NF-κB pathway inhibition, and upregulating osteogenic genes, GelMA-Z effectively treated periodontitis and accelerated periodontal tissue regeneration. In addition, the released Mg^2+^ activated Ca^2+^ channels on the membrane of osteoblasts, accelerating local calcium deposition. Western blot results showed a significant increase in OCN, Runx2, and ALP expression in induced BMSCs, indicating that both Mg^2+^ and exosomes exhibited significant bone induction effects.

The formation of new blood vessels (angiogenesis) is vital for bone repair because it supplies the oxygen, nutrients, and growth factors that new tissues need to heal. There are various strategies to promote angiogenesis, including accelerating extracellular matrix remodeling, promoting endothelial cell migration, and enhancing the expression of angiogenic factors. The most commonly used strategy is to stimulate the expression of angiogenic factors. For example, several metal ions, such as Co^2+^ and Ni^2+^, mimic the intracellular hypoxic conditions of vascular endothelial cells and promote the expression of VEGF (54, 55). Besides metal ions, drugs and endogenous signaling molecules are effective in increasing the levels of related angiogenic factors. MOFs can release metal ions and transmit signaling molecules to promote angiogenesis, thereby demonstrating the potential to promote periodontal tissue regeneration. By encapsulating cytokines such as VEGF and exosomes, MOFs can also protect these factors from degradation and enable their sustained release at bone defect sites. This controlled release ensures the presence of angiogenic signals over a long period of time, promoting a mature and fully functional vascular network.

Chronic periodontitis in diabetic patients exacerbates the destruction of local periodontal soft tissues and accelerates the resorption of hard tissues. Qu et al. found that magnesium-based metal-organic frameworks (Mg-MOF) exhibited significant efficacy in promoting tissue regeneration in diabetic periodontitis (56). The Mg-MOF was combined with glucose oxidase (GOX) to form a GOX/Mg-MOF composite system, which was integrated into double-layer microneedles (d-MNs) to construct a therapeutic platform. The GOX/Mg-MOF could reduce local blood glucose levels, scavenge reactive oxygen species, and the released Mg^2+^ could promote angiogenesis. The basal layer, composed of GelMA composite with nano-hydroxyapatite (nHA), closely adhered to the alveolar bone surface, induced osteoblast differentiation, upregulated osteogenic genes, and increased ALP activity and the number of mineralized nodules. *In vivo* experiments showed that after 28 days of treatment, the density of collagen fibers in periodontal tissues increased by 55%, the alveolar bone volume fraction rose from 23 to 32%, the CEJ-ABC distance shortened by 35%, and the thickness and number of bone trabeculae increased by 28 and 30%, respectively. Moreover, Mg^2+^ could regulate the polarization of macrophages toward the M2 phenotype and reduce the secretion of pro-inflammatory factors. Liang et al. constructed ZIF-8 loaded with dexamethasone (DEX@ZIF-8) to promote bone regeneration. ZIF-8 were coated with stem cell membranes (SCM), endowing them with the ability to reduce immunogenicity and target BMSCs through homologous binding. The RNA sequencing results also showed that DEX@ZIF-8-SCM upregulated transcription factors such as Osterix and Smad4 to enhance osteogenic differentiation of BMSCs (57).

In addition to providing bone-inducing drugs, MOFs can also transport specific signaling molecules to promote osteogenesis. ZIF-8 loaded with miRNA-21 and miRNA-5106 were able to accelerate vascularized bone regeneration (58). Nanoscale ZIF-8 not only protected miRNA through encapsulation but also promoted effective delivery of miRNA into cells through endocytosis. In particular, miRNA-5106 played a dominant role in osteogenic induction and promoting osteogenic differentiation of BMSCs by activating Wnt/β-catenin and TGF-β/Smad pathways (59).

In summary, MOF-based materials have primarily focused on enhancing bone properties in periodontitis management, effectively mitigating alveolar bone loss induced by excessive inflammatory responses. While research into their application in periodontitis treatment remains somewhat limited, advancements in material engineering are anticipated to drive the development of diverse high-performance MOF-derived materials, with promising extensions to periodontitis therapy. Collectively, through multifaceted mechanisms-including antibacterial activity, immunomodulation, promotion of osteogenic differentiation, regulation of relevant signaling pathways, and antioxidant stress-various MOFs play a pivotal role in promoting tissue regeneration during periodontitis treatment (Table 4), thereby offering novel strategies and directions for addressing this prevalent oral condition.

**TABLE 4:** Comparison of different MOF-based therapeutic strategies.

Strategy	Primary goal	Core mechanisms	Representative MOF systems
Antibacterial strategy	Eradicate pathogenic bacteria and disrupt biofilms.	Intrinsic bactericidal ion release; PTT/PDT; CDT; Delivery of antibiotic drugs.	CuTCPP-Fe2O3; MPB-BA; ICG@Uio-66-UBI; Ag@MOF
Regenerative strategy	Promote the regeneration of periodontal tissues (bone, PDL, cementum).	Release of osteoinductive ions (Zn^2+^, Mg^2+^, Ca^2+^); Delivery of growth factors (BMP-2), miRNAs, or exosomes; Scaffolding to support cell adhesion and differentiation; Promoting angiogenesis.	ZIF-8/GelMA-Z; Mg-MOF/PLGA/Exo-Mg-GA; miRNA-21/@ZIF-8; GOX/Mg-MOF (for diabetic periodontitis)
Multifunctional strategy	Integrated therapy: simultaneous antibacterial, anti-inflammatory, and regenerative actions.	Combination of the above mechanisms in a single platform; Use of smart, responsive systems (e.g., pH, ROS, enzyme-sensitive) for controlled release; Active immunomodulation to resolve inflammation and initiate healing.	CSBDX@MOF (Hydrogel-MOF composite); HMUiO-66-NH_2_ (ROS-scavenging and osteogenic); DEX@ZIF-8-SCM (Anti-inflammatory and targeted regenerative)

## 3 Multifaceted mechanisms of action of MOFs in periodontitis therapy

### 3.1 Intrinsic release of ligand components

MOFs achieve sustained ion release through their porous coordination frameworks, which undergo controlled degradation under physiological conditions, such as via local pH variations or ion exchange. This process is governed by the intrinsic stability, porosity, and metal-ligand bond strength of the MOF, collectively modulating the elution kinetics (60–62). Structurally incorporated metal ions, including Zn^2+^, Cu^2+^, and Ag^+^ known for their potent bioactivities, are released progressively (63, 64). This controlled elution profile prevents the rapid ion burst and aggregation that typically induce cytotoxicity and inflammatory responses, thereby ensuring long-term therapeutic efficacy with minimized adverse effect (65).

Beyond direct antibacterial effects, these released metal ions play sophisticated and multifaceted roles in immunomodulation and osteogenesis by regulating specific cellular signaling pathways and transcriptional programs. Owing to the unique ability of MOFs to donate essential metal ions in a sustained manner, several osteoinductive metal ions (e.g., Mg^2+^, Zn^2+^) have been demonstrated to not only exert antibacterial effects but also reduce ROS levels—exhibiting robust anti-inflammatory activity—and play critical roles in the activation of BMSCs and osteoblasts (66–69).

Zn^2+^ function as critical signaling modulators. The antibacterial impact of zinc ions, as demonstrated in ZIF-8@Rutin which significantly suppressed bacterial growth *in vitro* (63), is due to their controlled release and the subsequent formation of ROS. Moreover, Zn^2+^ suppresses the activation of the NF-κB pathway, leading to the downregulation of pivotal pro-inflammatory cytokines. Concurrently, it promotes macrophage polarization toward the anti-inflammatory M2 phenotype, facilitating inflammation resolution. Osteogenically, Zn^2+^ activates the MAPK/ERK signaling cascade, which upregulates the expression of master osteogenic transcription factors Runx2 and Osterix, thereby enhancing the synthesis of bone matrix proteins (19, 53).

Cu^2+^ exhibit a dual functionality. Liu et al. confirmed the remarkable antibacterial efficacy of a copper-based MOF (Cu MOF-1), where the liberated ions impaired bacterial membrane stability and interfered with intracellular proteins and deoxyribonucleic acid (DNA) (31). While capable of catalyzing ROS generation for antibacterial purposes, Cu^2+^ also possesses potent pro-angiogenic properties. It mimics intracellular hypoxia, stabilizing hypoxia-inducible factor-1α (HIF-1α), which subsequently upregulates VEGF expression, a process crucial for supporting new bone formation.

Ag^+^, renowned for their broad-spectrum antimicrobial efficacy as shown in a PLGA/HA scaffold with an antibacterial efficacy of 87.8% (70), also demonstrate immunomodulatory potential at controlled concentrations. The controlled release of silver ions effectively destabilizes cell membranes and stimulates ROS generation. Furthermore, they can attenuate excessive inflammatory responses by modulating key signaling pathways in macrophages, thereby mitigating collateral tissue damage.

Mg^2+^ are instrumental in orchestrating the bone regeneration microenvironment. The bactericidal effect of Mg-MOF has been attributed to its organic constituents (56). More significantly, Mg^2+^ contributes to immunomodulation by driving macrophage polarization from the pro-inflammatory M1 to the pro-repair M2 state. In osteoblasts, Mg^2+^ released from Mg-MOFs activates the Notch and PI3K/AKT pathways and stimulates calcium channels, thereby promoting osteogenic differentiation of BMSCs and accelerating mineral deposition (46, 53).

The integration of MOF-based materials into hydrogels or other carriers further expands their utility, enhancing retention and controlled release at the periodontal site (64). Collectively, the intrinsic release of these metal ions from MOFs enables a tripartite therapeutic strategy: direct pathogen eradication, precise immunomodulation, and active promotion of bone regeneration. This multifaceted mechanism, leveraging tunable degradation for controlled, long-term delivery, effectively circumvents the limitations of conventional ion-releasing materials and establishes MOFs as a superior platform for managing complex chronic conditions like periodontitis.

### 3.2 Catalytic activity

MOFs demonstrate remarkable versatility in ROS regulation, exhibiting both ROS-scavenging and ROS-generating capabilities through distinct mechanisms. As efficient nanozymes, MOFs mimic natural enzyme activities, particularly peroxidase-like properties, enabling their application in chemodynamic therapy where they convert endogenous H_2_O_2_ into highly bactericidal ⋅OH through Fenton and Fenton-like reactions (71–74). This iron-catalyzed process represents an autocatalytic mechanism that operates without requiring external energy input, making MOFs particularly suitable for antibacterial applications (75, 76).

The antibacterial efficacy of MOF-mediated ROS generation operates through multiple pathways: excessive intracellular ROS accumulation directly disrupts cellular integrity by inducing DNA single-strand breaks and impairs bacterial viability through oxidation of critical biomolecules including nucleic acids, proteins, and lipids (77, 78). This cumulative oxidative damage ultimately suppresses bacterial metabolism and proliferation, leading to irreversible inactivation (77, 78). For instance, trimetallic MOF nanosheets have demonstrated exceptional catalytic activity in reducing H_2_O_2_ to generate ⋅OH, showing outstanding antibacterial effects against various pathogens including *E. coli* and methicillin-resistant S. *aureus* while promoting wound healing in infection models (79).

Complementing their ROS-generating functions, certain MOF systems exhibit sophisticated ROS-scavenging capabilities through specific catalytic mechanisms. HMUiO-66-NH_2_ nanoparticles possess peroxidase-like activity that catalyzes H_2_O_2_ decomposition without generating ⋅OH via the Fenton reaction, enabling selective clearance of excess H_2_O_2_ while preserving physiological signaling functions (80). Similarly, Zn/Co-MOF utilizes Co^2+^/Co^3+^ valence transitions to mimic superoxide dismutase and catalase cascade catalysis, effectively eliminating O_2_^–^ and H_2_O_2_ with substrate selectivity toward inflammation-related ROS species (17). The Mg-GA MOF employs an alternative mechanism, relying on phenolic hydroxyl groups from gallic acid ligands as hydrogen donors to scavenge free radicals through non-metallic-center electron transfer, thereby preventing secondary oxidative damage (14).

The spatial and temporal control of ROS generation is achieved through strategic material design. Systems such as ICG@Uio-66-UBI generate ROS via photosensitizer activation under near-infrared light while producing negligible ROS in darkness, achieving precise spatiotemporal control (41). Ag@MOF exploits nanoscale dimensions (approximately 5.5 nm) to promote bacterial internalization and localized ROS generation, while structural stability and encapsulation within sustained-release carriers limit off-target toxicity in host tissues (30).

To intelligently balance these dual functionalities, MOFs are integrated with responsive carrier systems. Mg-GA MOF encapsulated within pH/ROS dual-sensitive hydrogels enables preferential release in periodontitis regions characterized by high ROS and alkaline pH, spatially segregating antioxidant and osteogenic functions (14). Zn/Co-MOF employs thermosensitive gels to achieve controlled ion release, maintaining osteogenic activity while mitigating cellular Co^2+^ accumulation risks (17).

**FIGURE 4:**
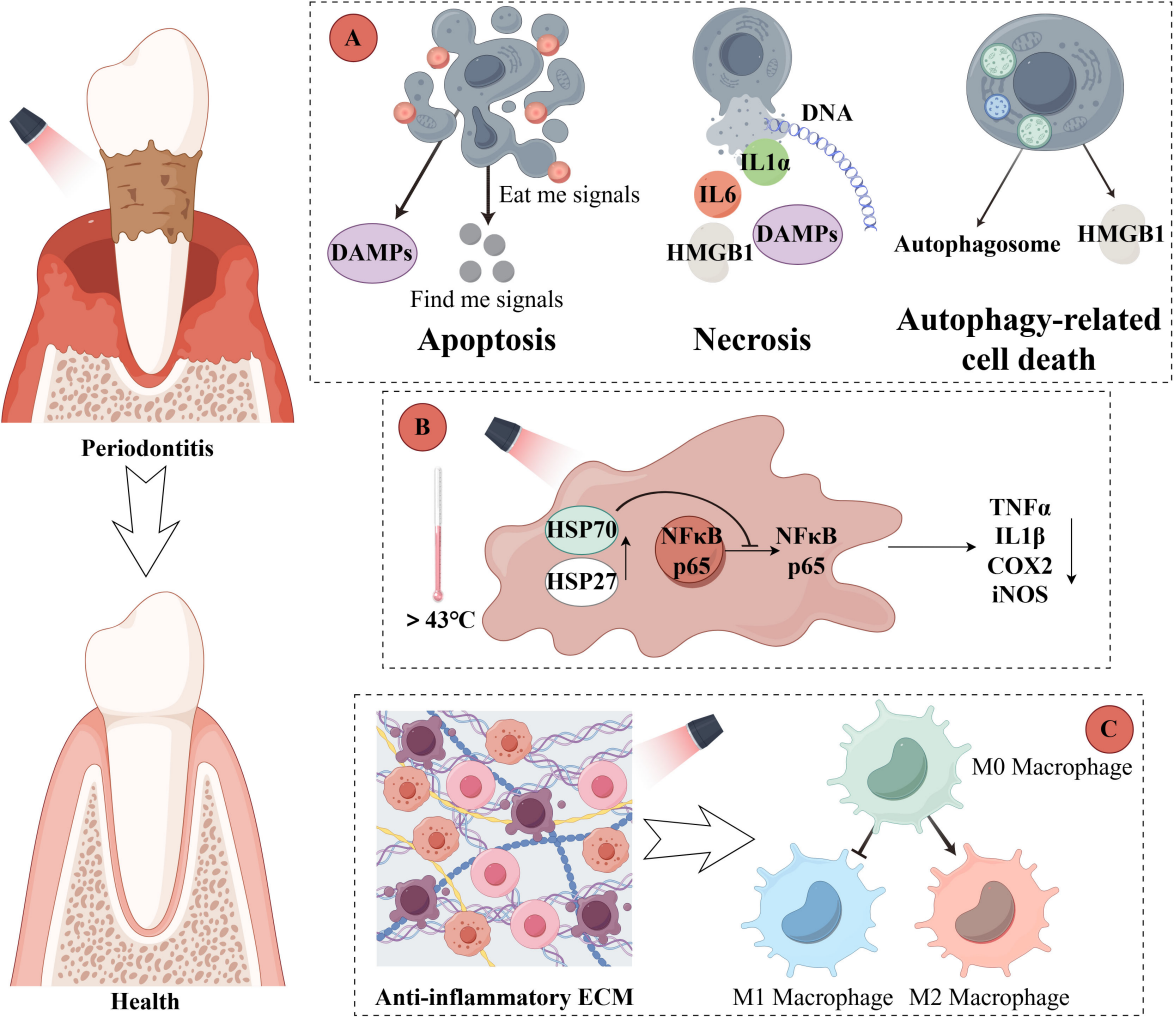
MOF-mediated photodynamic and photothermal therapy in periodontitis. **(A)** Cell death modalities and release of damage-associated molecular patterns (DAMPs) in periodontal lesions. **(B)** Anti-inflammatory effects of MOF-induced photothermal heating. **(C)** Modulation of the immune microenvironment by MOF-based phototherapy. The figure was created with Figdraw (www.figdraw.com).

These MOF systems demonstrate favorable biosafety profiles at optimized doses, showing no significant hemolysis, apoptosis, or histopathological damage (17, 80). Advanced systems further minimize off-target effects through targeting modifications and localized release mechanisms (35). The mechanisms and spatiotemporal controllability of MOF-mediated PTT and PDT are summarized in Figure 4. Compared to conventional therapies, MOF-mediated chemodynamic therapy exhibits superior specificity and fewer side effects, as the ROS generation process responds to inflammatory microenvironments while causing minimal damage to normal tissues.

In summary, MOFs achieve sophisticated spatiotemporal control through composition tuning, structural functionalization, and carrier integration, enabling context-dependent switching between ROS generation for antibacterial action and ROS scavenging for oxidative stress alleviation. This dynamic regulation capability positions MOFs as promising platforms for developing targeted therapies against inflammatory tissue defects and bacterial infections.

### 3.3 Photothermal therapy and photodynamic therapy

MOFs have demonstrated potent antibacterial effects through PTT and PDT, showing promising potential for the treatment of periodontitis (Figure 4) (81). Compared to conventional antibiotic therapies, PTT and PDT are less likely to induce bacterial resistance in periodontal pathogens. Furthermore, the light sources employed in photothermal antibacterial therapy typically exhibit deeper tissue penetration due to their specific wavelength characteristics, enabling more effective access to deep periodontal pockets (82, 83). Importantly, the precise spatial and temporal control of irradiation parameters allows for minimized side effects on adjacent healthy tissues.

The underlying mechanism primarily involves Fenton-like reactions, where nanoengineered MOFs serve as catalysts that are selectively delivered to inflammatory sites. These catalysts promote the generation of high concentrations of hydroxyl radicals ( ⋅OH) and ROS through Fenton or Fenton-like reactions (84). The resulting oxidative stress induces irreversible damage to critical bacterial components including lipids, DNA, and proteins, ultimately leading to inflammatory cell death via multiple pathways such as apoptosis, autophagy, and necrosis. This approach offers superior inflammatory specificity compared to other ROS-based therapies, effectively minimizing oxidative damage to normal tissues (79).

Notably, when PDT is combined with other therapeutic modalities, both the efficacy and sensitivity of anti-inflammatory effects are significantly enhanced (85). Such combinatorial strategies can overcome the limitations of monotherapies, potentially achieving more robust and broadly applicable anti-inflammatory outcomes (78).

Photodynamic and photothermal therapy depend on external stimuli and demonstrate superior selectivity and controllability compared to automatic catalytic systems. MOFs are widely used in fields such as photosensitizers and photothermal agents. The attributes above have prompted the investigation of MOFs in treating bacterial infections and have yielded encouraging therapeutic outcomes (86).

Photodynamic systems, comprising light, photosensitizers, and tissue oxygen, are anticipated to emerge as attractive alternatives for antibacterial applications. Upon exposure to sunlight, the photosensitizer undergoes a step-by-step activation process, changing from its initial condition to a state of singlet excitation and, subsequently, triplet excitation (87). The triple-excited photosensitizer produces ROS by forming free radicals through electron or hydrogen transfer and interaction with ground-state molecular oxygen to produce highly reactive singlet oxygen species (88). The generated ROS can disturb cellular architecture and impede fundamental processes, ultimately resulting in bacterial death. Although photodynamic therapy possesses favorable attributes such as non-invasive and high specificity, its efficacy depends on the availability of appropriate photosensitizers. Fortunately, MOF-based materials have emerged as intriguing candidates owing to their tunable semiconductor characteristics, simplicity of modification, and excellent biocompatibility. These findings highlight the promise of MOFs as effective photosensitizers for photodynamic therapy, enabling the *in situ* generation of ROS under optical excitation within the physiological oxygen microenvironment (88).

Li et al. used the synthesized CuTCPP-Fe_2_O_3_ with the ethylene glycol matrix to create a localized ointment for treating periodontitis (31). When exposed to light radiation, the photodynamic effect combined with copper and iron ions causes harm to the structure and function of the cell, including alterations in cell membrane permeability and deactivation of proteins. It demonstrated that the modified MOF efficiently suppressed the growth of P. gingivalis, F. nucleatum, and S. aureus with an antibacterial effect of over 99%. Furthermore, investigations *in vivo* verified that when compared to conventional antibiotic therapy, the photodynamic ion system offered efficient antibacterial treatment, alleviated inflammation, promoted angiogenesis, and dramatically enhanced the prognosis of periodontitis. Photothermal therapy relies on the energy conversion properties of photothermal agents (PTA). PTA typically efficiently transforms absorbed energy into thermal energy when subjected to electromagnetic radiation, including radio frequency, microwave, near-infrared radiation, or visible light. It has recently garnered significant interest and gained traction in the medical profession, showing great potential for further advancement (89). Photothermal and photodynamic therapy differ in their utilization of ROS created during light exposure. Photothermal therapy achieves sterilization by raising the temperature in a specific area (90). Elevated temperature can result in the deactivation of bacteria by causing harm to their membranes and denaturing their proteins. Nevertheless, it is imperative to avoid exposing the surrounding healthy tissues to extreme temperatures that may cause damage. In order to tackle this problem, scientists have investigated the combination of photothermal therapy with other methods, such as chemotherapy, hemodynamic therapy, and photodynamic therapy, so as to achieve a more powerful antibacterial impact. These multimodal techniques offer potential therapeutic benefits by decreasing reliance on photothermal agents and laser intensity. The development of MOF materials provides a suitable platform for implementing these strategies. Accumulated evidence indicates that MOF-based PTA has promising potential for antibacterial applications (91).

Tian et al. introduced a platform using MOF to encapsulate BA onto mesoporous MPB nanoparticles (16). MBP functioned as a PTA and exhibited its antibacterial properties when exposed to light. BA removed intracellular ROS and stimulated the polarization of macrophages toward the M2 phenotype. It demonstrated that the material, built on a MOF, effectively suppressed the proliferation of gingival and nuclear filamentous bacteria when subjected to near-infrared radiation. The antibacterial efficacy of this material exceeded 99% against both types of bacteria. BA-MPB was shown to modulate the nuclear factor erythroid 2-related factor 2/nuclear factor kappa-light-chain-enhancer of activated B cells (Nrf2/NF-κB) signaling pathway, effectively suppressing the inflammatory response. Further *in vivo* findings demonstrated that BA-MPB had superior effectiveness in decreasing periodontitis in rats compared to conventional antibiotics. It was due to the antibacterial and immune regulatory properties of BA-MPB.

Since the initial introduction in 1989 by Yumita et al., Sogdynamic therapy has been considered a promising therapeutic approach. Sonodynamic therapy is an emerging antibacterial method based on ultrasound irradiation. Compared with photodynamic therapy and photothermal therapy, sonodynamic therapy exhibits superior ultrasound penetration capability, making it suitable for treating deep-seated cancers and infectious diseases (92). It is indicated that the antibacterial properties of hyperkinetic therapy may be attributed to the generation of ROS. When ultrasound is used on the target tissue, microbubbles in the surrounding fluid are formed. The fast generation and subsequent collapse of these bubbles result in the release of energy, causing the sonosenitizer to transition into an excited state and generate ROS. Currently, the precise mechanism behind sonoluminescence remains uncertain, although new studies indicate that it may encompass blackbody radiation, bremsstrahlung radiation, recombination radiation, or a mix of these phenomena (92, 93).

To enhance the effectiveness of MOF-based materials against bacteria, scientists have investigated the creation of MOFs with combined antibacterial mechanisms. As an illustration, Zhang and his colleagues created a Zn MOF that incorporated the photosensitizer chloroe6 (Ce6), named Ce6@MOF (94). It was incorporated into the thermosensitive hydrogel to form an antibacterial wound dressing, expressed as Ce6@MOF-Gel. It demonstrated that the antibacterial activity was at a relatively low potency level when laser irradiation was absent. When exposed to laser radiation, Ce6@MOF-Gel exhibited high antibacterial efficacy, with an average survival rate of 1.21 ± 0.15%. It released zinc ions and produced ROS through the photodynamic mechanism when exposed to laser light, demonstrating the antibacterial effect. Further, *in vivo* experiments revealed that it significantly accelerated wound healing in a rat model infected with Pseudomonas aeruginosa. Zheng et al. created a MOF based on ceriumIII/ceriumIV, which was named CeTCPP Au, with excellent dynamic antibacterial activity (95). When exposed to ultrasonic radiation, gold nanoparticles caused a modification in the oxidation state of cerium in CeTCPP by capturing electrons and induced alterations in coordination and impairments in cerium nodules. The presence of the uneven structure impedes the recombination of electron pores and enhances the generation of ROS. It was important to mention that when exposed to ultrasound, the antibacterial effectiveness of CeTCPP Au against S. aureus was 99.73 ± 0.05%, while the effectiveness against E. coli was 99.16 ± 0.29%. Furthermore, CeTCPP Au exhibited favorable outcomes in managing osteomyelitis induced by *Staphylococcus aureus* infection in rats, highlighting the potential of MOF as a sonosensitizer for treating profound infections.

The integration of PTT and PDT, as exemplified by the aforementioned systems, effectively transforms MOFs from static drug carriers into externally activatable and spatially precise therapeutic devices. This capability for on-demand, targeted antibacterial and anti-inflammatory action, with minimal risk of inducing resistance, constitutes a novel advance over both conventional antibiotics and simple ion-releasing biomaterials, paving the way for more intelligent and controllable periodontal therapies. However, there is limited research on using MOFs as sonosensitizers in antibiotic therapy. To determine the effectiveness and safety of MOFs as sonosensitizers, further comprehensive and systematic research is needed to pave the way for the future application of MOFs in clinical settings.

### 3.4 Extraneous component release system

**FIGURE 5:**
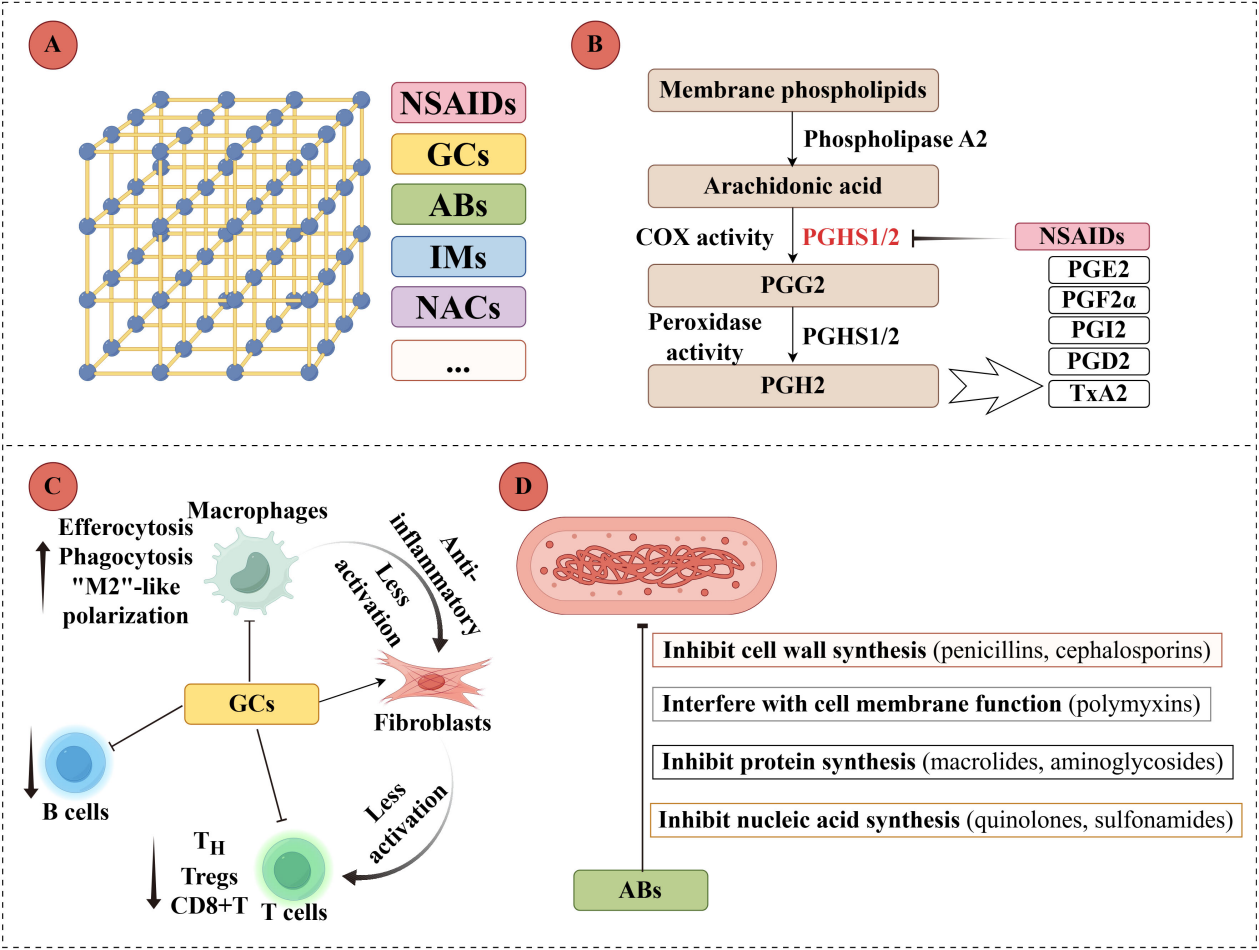
MOF-mediated release of exogenous components for periodontitis treatment. **(A)** MOFs as nanocarriers encapsulating diverse drug molecules (NSAIDs, glucocorticoids, antibiotics, immunomodulators, antioxidants). **(B)** Mechanism of NSAIDs released from MOFs inhibiting cyclooxygenase (COX) activity. **(C)** Regulation of immune cells by glucocorticoids (GCs) released from MOFs to suppress periodontal inflammation. **(D)** Core antibacterial mechanisms of antibiotics (ABs) released from MOFs. The figure was created with Figdraw (www.figdraw.com).

Furthermore, MOFs serve as a drug delivery system for periodontal pockets, **a**s schematically depicted in Figure 5. Their engineered porosity allows for the encapsulation and controlled release of a diverse range of exogenous therapeutic agents, including non-steroidal anti-inflammatory drugs (NSAIDs), glucocorticoids (GCs), antibiotics, and immunomodulators (96). In an investigation, the medication DEX, a type of steroid with anti-inflammatory and immunosuppressive properties, was included in ZIF-8. This specific nanoparticle could achieve precise release of DEX, decrease the expression of matrix metalloproteinase-9 produced by macrophages, and diminish the infiltration of inflammatory cells in periodontium (8). Tian et al. created a nanoparticle named MPB-BA, consisting of mesoporous MPB and the antioxidant BA (16). It demonstrated that it is highly effective in converting light into heat energy. When exposed to NIR irradiation, the rise in local temperature caused the separation of MPB-BA connections, enabling the precise release of the loaded BA. Experimental findings indicated that MPB-BA enhanced the phosphorylation and movement of nuclear factor 2-associated factors within red blood cells. It then increased the expression of antioxidant genes to protect macrophages from oxidative stress and transitioned them from the M1 to the M2 phenotype. MPB-BA effectively decreased alveolar bone loss in rats with experimental periodontitis, and its effectiveness was superior to that of minocycline hydrochloride. It highlights the potential applications of materials based on MOFs and provides possible solutions with antibacterial mechanisms for infection treatment.

### 3.5 Comparative analysis of advanced MOF platforms

The transition from mechanistic concept to therapeutic application is demonstrated by the leading MOF designs compiled in Table 5. This analysis directly correlates rational design choices with enhanced therapeutic outcomes, providing definitive evidence of their improved efficacy, controlled release profiles, and multifunctional capacity compared to conventional single-action modalities. Collectively, these platforms demonstrate distinct advantages over conventional therapies, which can be summarized as follows:

**TABLE 5:** Comparative analysis of advanced MOF designs: mechanistic insights and advantages over conventional treatments.

MOFs system	Ligand	Loaded agent	Delivery method	Key findings (antibacterial/ osteogenic)	Advantages over conventional methods	*In vitro/in vivo* results	Limitations
ZIF-8	Zn^2+^/2-Methylimidazole	DEX	Nanocomposite Hydrogel (GelMA)	Antibacterial: Significant inhibition of *P. gingivalis* and *S. aureus*. Anti-inflammatory/Osteogenic: Sustained release of Zn^2+^ and DEX for > 7 days; significantly downregulated IL-6, TNF-α; upregulated Runx2, OCN, ALP expression. Promoted periodontal bone regeneration in rat models	Controlled Release: Provides sustained anti-inflammatory stimulation for over 7 days, unlike the burst release of free DEX (> 80% within 24 hrs). Multifunctionality: A single platform simultaneously achieves antibacterial (Zn^2+^), anti-inflammatory (DEX), and pro-osteogenic (Zn^2+^) effects, whereas traditional methods often require combined formulations	*In vitro*: Effective drug encapsulation and release (e.g., doxorubicin); *In vivo*: Good biocompatibility and tumor targeting in mouse models	Potential zinc toxicity; poor stability under physiological conditions; incomplete drug release
CuTCPP-Fe2O3	Cu^2+^, Fe^3+^/TCPP	(Inherent photosensitizer)	Photo-responsive Ointment (PEG matrix)	Antibacterial: > 99% inhibition against *P. gingivalis*, *F. nucleatum*, and *S. aureus* under PDT. Anti-inflammatory/Osteogenic: Significantly downregulated iNOS, COX-2; upregulated VEGF, CD31. Effectively promoted bone regeneration in a rat model, outperforming antibiotic groups.	Synergistic and targeted therapy: combines PDT with metal ion therapy, enabling spatiotemporally precise treatment via light irradiation, avoiding side effects of systemic antibiotics. Low Resistance Risk: Multi-mechanistic antibacterial action (ROS + ion interference) reduces the likelihood of resistance development compared to single-target conventional antibiotics.	*In vitro*: ROS production under light, effective cancer cell killing; *In vivo*: Enhanced tumor accumulation and therapeutic efficacy in rat models via magnetic targeting	Limited light penetration depth for deep tumors; potential copper and iron toxicity; complex synthesis
MPB-BA	Fe^3+^/Ferrocyanide	BA	Nanoparticles (topical application)	Antibacterial: NIR-induced photothermal heating to 88.8°C, synergizing with BA, resulting in > 99% antibacterial rate against pathogens. Immunomodulation: Scavenged ROS, promoted macrophage polarization from M1 to M2 phenotype, reduced CEJ-ABC distance.	On-demand delivery and immunomodulation: NIR triggers drug release and photothermal effect, allowing “on/off” control. Actively modulates the immune microenvironment, a feature absent in traditional antibiotics and simple PTT. Reduced Dosage: Single treatment efficacy surpassed that of continuously administered minocycline hydrochloride.	*In vitro*: Demonstrated drug loading and release capabilities; *In vivo*: Limited data, may show preliminary biocompatibility in mouse models	Mechanism not well-defined; potential stability issues under physiological conditions; unknown toxicity; limited research
Mg-GA MOF	Mg^2+^/GA	(GA as ligand); exosomes	Composite hydrogel; PLGA electrospun scaffold	Antibacterial/Anti-inflammatory: Synergistic antibacterial effect of Mg^2+^ and GA; inhibited NF-κB pathway, reducing iNOS, COX-2, IL-6. Osteogenic/Angiogenic: Promoted macrophage M2 polarization, activated RUNX2, upregulated VEGF/CD31. Significantly enhanced new bone formation in a rat calvarial defect model	Bioactive Ligand: The ligand GA itself possesses anti-inflammatory and antioxidant activities, making the MOF framework intrinsically therapeutic, unlike traditional inert carriers. Microenvironment Remodeling: Systematically modulates immune and osteogenic microenvironment via Mg^2+^ release and exosome delivery, exceeding the mere “filling” function of traditional bone grafts	*In vitro*: High antioxidant activity, protects cells from oxidative damage; *In vivo*: Reduces oxidative stress-related symptoms in mouse models	Magnesium-based MOFs prone to degradation under physiological conditions; limited drug loading capacity; lack of targeting
DEX@ZIF-8-SCM	Zn^2+^/2-Methylimidazole	DEX	Stem cell membrane (SCM) coated nanoparticles	Targeting/Osteogenic: SCM coating enabled active targeting to BMSCs. RNA sequencing showed upregulation of Osterix and Smad4, significantly enhancing osteogenic differentiation of BMSCs	Active Targeting: The stem cell membrane camouflage technology allows specific targeting of BMSCs, increasing drug enrichment at the bone defect site and reducing off-target effects, a level of precision unattainable with traditional local delivery	*In vitro*: Effectively inhibits macrophage inflammatory response; *In vivo*: Enhanced targeting and anti-inflammatory effects in mouse inflammation models	Complex preparation, high cost; limited source of stem cell membranes; long-term safety not fully studied

a. Integrated functionality and synergistic therapy. Unlike conventional single-action treatments (e.g., mechanical debridement, local antibiotics), MOFs such as ZIF-8 and Mg-GA MOF integrate antibacterial, anti-inflammatory, and pro-osteogenic functions into a unified platform. This integration enables synergistic interactions, yielding a therapeutic outcome that is superior to the sum of individual effects; b. Controlled and on-demand drug release. MOF-based systems (e.g., MPB-BA, ZIF-8@DEX) allow for precise manipulation of drug release kinetics. This ranges from sustained release to stimuli-triggered (e.g., light, pH) on-demand delivery. This capability mitigates the initial burst release and associated toxicity of conventional formulations, thereby significantly reducing the total dosage required and the frequency of administration; c. Microenvironment responsiveness and immunomodulation. Advanced MOF designs (e.g., CuTCPP-Fe2O3, MPB-BA) function beyond mere pathogen eradication. They actively sense and remodel the inflammatory periodontal microenvironment, for instance, by scavenging excessive ROS and promoting macrophage polarization from the pro-inflammatory M1 phenotype to the pro-healing M2 phenotype. This sophisticated immunomodulatory capacity is absent in traditional antibiotic regimens; d. Reduced risk of antimicrobial resistance. MOFs exert antibacterial effects through multi-mechanistic actions, including metal ion release, ROS burst, and physical disruption. This multi-target approach presents a significant barrier to bacteria, which typically develop resistance through single-step mutations, thereby offering a promising strategy to combat the growing crisis of antibiotic resistance.

In summary, through rational selection of metal/ligand and functionalization, MOFs transcend the role of simple drug carriers to become intelligent platforms integrating diagnostic and therapeutic functions. They demonstrate significant potential to surpass conventional methods in terms of controllability, synergy, and therapeutic efficacy. A comparative overview of MOFs against other localized nanoplatforms is summarized in Table 6 (42, 97–100).

**TABLE 6:** Comparison of MOFs with mainstream nanoplatforms in core properties and applications.

Comparison dimension	MOFs	Liposomes	Dendrimers	Mesoporous silica
Carrier structural features	Porous crystals composed of metal ions/clusters and organic ligands; adjustable pore size (0.5–10 nm); ultra-high specific surface area (500–5,000 m^2^/g)	Closed vesicles formed by phospholipid bilayers with an aqueous core; particle size (20–500 nm); no fixed pore size	Highly branched spherical macromolecules with abundant surface active groups; particle size (1–10 nm); precisely controllable structure (defined by generation)	Ordered mesoporous channel structure (pore size 2–50 nm); good channel connectivity; easily modifiable surface silanol groups
Surface modification difficulty	Easy	Moderate	Easy	Easy
Loading capacity and mechanism	High	Moderate	Moderate	High
Responsive release performance	Excellent	Moderate	Moderate	Moderate
Biocompatibility and safety	Moderate	Excellent	Moderate	Excellent
Core functional advantages	1. Porous structure and ultra-high specific surface area enable loading capacity far exceeding traditional carriers; 2. High structural designability supports multi-functional integration (e.g., theranostics); 3. Multi-stimuli responsiveness adapts to complex physiological microenvironments	1. Biomimetic membrane structure facilitates cell fusion and enhances intracellular cargo delivery efficiency; 2. Enables co-delivery of hydrophilic/hydrophobic drugs; 3. Mature clinical translation (e.g., doxorubicin liposomes are clinically approved)	1. Precisely controllable molecular structure allows quantitative modification of targeting/functional groups; 2. High surface charge density facilitates binding to negatively charged biomolecules (e.g., DNA)	1. Stable mesoporous structure resists acid/alkali and high temperatures, adapting to various preparation processes; 2. Uniform pore size enables homogeneous cargo release; 3. Excellent targeting after surface modification
Main limitations	1. Poor water stability of some MOFs leads to premature dissociation in physiological environments; 2. Potential toxicity of metal ions and unclear *in vivo* metabolism mechanisms; 3. High cost for large-scale preparation	1. Poor stability (prone to fusion/leakage; special conditions required for long-term storage); 2. Low drug loading capacity (unsuitable for high-dose drug delivery); 3. Easily cleared by the mononuclear phagocyte system (MPS)	1. Complex synthesis steps and high difficulty in large-scale preparation; 2. Limited internal cavity (weak loading capacity for macromolecular drugs like proteins); 3. Long-term *in vivo* retention may trigger inflammatory responses	1. Limited pore size adjustment range (incompatible with ultra-small or ultra-large cargo molecules); 2. Pores easily clogged, affecting release efficiency; 3. Lack of active responsiveness, making precise release difficult
Typical application scenarios	1. Tumor multi-modal theranostics (e.g., loading chemotherapeutic drugs + fluorescent imaging probes); 2. Antibacterial field (loading antibiotics + reactive oxygen species generation); 3. Enzyme immobilization and catalysis	1. Drug/gene delivery (e.g., siRNA/mRNA delivery); 2. Vaccine carriers (e.g., mRNA lipid nanoparticles for COVID-19); 3. Local drug delivery (e.g., dermal/ocular delivery)	1. Targeted drug delivery (e.g., tumor-targeted chemotherapy); 2. Gene therapy (e.g., non-viral gene vectors); 3. Biosensors (e.g., molecular recognition)	1. Controlled drug release (e.g., antibiotic sustained release in bone tissue engineering); 2. Adsorption and separation (e.g., heavy metal ion removal); 3. Catalyst carriers

## 4 Clinical translation challenges and prospects of MOF-based systems

The translation of MOFs from promising *in vitro* and pre-clinical candidates to clinically approved therapies for periodontitis necessitates a critical appraisal of their translational readiness, safety profile, and manufacturing scalability. This section delineates the MOF systems closest to clinical application and systematically addresses the formidable challenges that must be overcome.

### 4.1 Dynamic oral Microenvironment: implications for MOF stability and efficacy

For MOFs to be used in patients, they must work effectively in the challenging environment of the mouth. This microenvironment presents interconnected challenges that can compromise MOFs stability, dictate drug release kinetics, and ultimately determine therapeutic efficacy. Saliva flow can quickly wash away MOFs that do not stick well to the gums, reducing their effectiveness at the target site, reducing the local therapeutic concentration and raising concerns about unintended systemic exposure (101, 102). Furthermore, the periodontal pocket pH can fluctuate from neutral to acidic in active disease due to bacterial fermentation. While this can be leveraged by pH-responsive MOFs like ZIF-8 for targeted drug release, prolonged acidic exposure may also accelerate framework degradation, leading to a premature burst release of metal ions and payload that could induce cytotoxicity and shorten the therapeutic window (102). The oral cavity is also rich in bacterial and host-derived enzymes (e.g., esterases, proteases) which can degrade vulnerable organic ligands, destabilizing the MOFs crystalline structure and compromising its controlled release function (35, 103). Beyond this, the periodontal biofilm acts as a metabolically active entity; bacteria can secrete chelating agents that compete for metal ions, potentially disassembling the MOFs from within, while the dense extracellular polymeric substance can hinder MOFs penetration and shield pathogens (104). To overcome these hurdles, the field is focusing on engineering robust MOFs formulations. Primary mitigation strategies include encapsulation in bioadhesive hydrogels (e.g., chitosan, GelMA) to enhance retention and provide a secondary release barrier (12, 14, 30), surface engineering with stealth molecules like PEG to improve enzymatic stability, and the rational design of MOFs using metal-ligand combinations with higher coordination strength and enzymatically resistant linkers. Addressing these microenvironmental factors through intelligent material design is not an optional refinement but a prerequisite for developing clinically viable MOF-based therapies for periodontitis.

### 4.2 Methodological heterogeneity and validation gaps

Beyond material-centric hurdles, the clinical translation of MOF-based periodontitis therapies is impeded by two fundamental methodological limitations. First, the field lacks standardized preclinical models. Studies employ highly heterogeneous experimental systems, including variable bacterial strains, divergent biofilm protocols, and disparate animal models (105, 106). This methodological variability prevents meaningful comparison between MOF platforms and obscures critical structure-activity relationships. Developing pathologically relevant standards that accurately recapitulate the human periodontal environment is therefore essential to generate clinically predictive data and advance lead candidates (107, 108).

Second, the evidence for key therapeutic mechanisms such as macrophage polarization requires more rigorous validation. Reported shifts from M1 to M2 phenotypes often derive from simplified *in vitro* systems or short-term animal studies relying on limited surface markers. These approaches do not adequately capture the spectrum of macrophage activation states present in human periodontal lesions (109). Furthermore, the long-term stability and functional impact of MOF-induced immunomodulation on periodontal regeneration remain poorly understood (101). Future studies should implement high-resolution techniques, such as single-cell RNA sequencing, to precisely define immune cell heterogeneity and functional responses in the healing periodontium.

### 4.3 MOF systems with clinical translation potential

Among the diverse MOF structures investigated, systems constructed from endogenous or low-toxicity metal ions exhibit the most immediate clinical potential. Iron-based MOFs [e.g., MIL-100(Fe)] and zirconium-based MOFs (e.g., UiO-66(Zr)) are front-runners due to their well-documented biocompatibility, structural stability, and capacity for controlled drug delivery (110). The degradation products of MIL-100(Fe) can integrate into the body’s natural iron metabolism, while the robustness of UiO-66(Zr) makes it a promising platform for theranostic applications. Furthermore, zinc-based ZIF-8 has been extensively explored in periodontal research, leveraging its pH-responsive degradation to simultaneously release antibacterial Zn^2+^ ions and encapsulated therapeutic agents within the inflammatory microenvironment (111). Although no MOF has been approved for periodontal use, the progression of Hf-based RiMO-301 into oncology clinical trials validates the principle that MOF-class materials can meet the stringent requirements for human testing (112).

### 4.4 Major regulatory, safety, and manufacturing barriers

The journey from laboratory to clinic for MOFs is impeded by a series of interconnected hurdles spanning regulation, safety, and manufacturing. The hybrid nature of MOFs complicates their regulatory classification, as they do not fit neatly into existing categories for drugs or medical devices. Agencies like the FDA and EMA currently lack specific guidelines for characterizing MOF-based therapeutics, particularly concerning their purity, pharmacokinetics, and long-term fate *in vivo* (110, 112). This ambiguity necessitates the establishment of standardized evaluation protocols, which is a critical and non-negotiable step for clinical advancement. The long-term systemic safety of MOFs remains a paramount concern, with potential toxicity being highly MOF-specific rather than intrinsic. Key issues include the leaching of metal ions, where even biocompatible metals (e.g., Zn, Fe) can provoke oxidative stress and cytotoxicity at high concentrations or upon rapid release from unstable frameworks (110, 113). Furthermore, the metabolic fate and potential toxicity of organic linkers are often underexplored. A critical balance must be struck between structural stability for sustained therapeutic action and biodegradability for safe clearance. While smaller nanoparticles (< 5–6 nm) may undergo renal clearance, larger particles are often sequestered in the liver and spleen, raising concerns about chronic inflammation or fibrosis if they are not efficiently broken down (110, 113). Zirconium-based MOFs, prized for their stability, are particularly scrutinized for their potential long-term accumulation. Manufacturing and scalability present another significant barrier, as reproducible, large-scale synthesis under Good Manufacturing Practice (GMP) standards is challenging. The common use of organic solvents, precise reaction conditions, and the need for stringent control over critical quality attributes (e.g., particle size, porosity, and surface chemistry) across batches complicate industrial production (112). Ensuring sterility and long-term shelf stability adds another layer of complexity to the manufacturing process, completing a triad of major translational bottlenecks.

### 4.5 Concluding perspectives and future pathways

In conclusion, while MOFs offer a uniquely versatile platform for addressing the multifactorial pathology of periodontitis, their clinical translation is not imminent. Uncritical claims of “excellent biocompatibility” or inherent superiority over traditional materials are unsubstantiated; instead, the bio-safety of each MOF must be empirically and rigorously validated. Future research must pivot from proof-of-concept efficacy studies to a dedicated focus on several key areas. There is a pressing need for rational material design, which prioritizes the development of biodegradable frameworks using endogenous metals and metabolizable ligands to enhance biosafety. Comprehensive safety assessments must be conducted, involving systematic long-term toxicology and pharmacokinetic studies in advanced animal models to thoroughly evaluate potential risks. Industrial collaboration is essential to develop scalable, green synthesis routes and address the myriad GMP-related challenges associated with production. Finally, proactive regulatory engagement is required, where researchers work closely with regulatory bodies to define the necessary benchmarks and standards for MOF-based therapeutics (110, 112). Only through such a concerted, interdisciplinary effort can the formidable gap between laboratory innovation and clinical deployment be bridged, ultimately unlocking the full potential of MOFs in periodontal care. In conclusion, the future of MOF-based periodontitis therapy lies not in further optimization of passive ion-release systems, but in the deliberate design of intelligent, multifunctional, and immunomodulatory platforms. Future research should be channeled toward these complex systems that can provide comprehensive solutions by integrating targeted antibacterial action, inflammation resolution, and guided tissue regeneration in a spatiotemporally controlled manner.

## 5 Conclusion

In conclusion, MOFs represent a promising next-generation therapeutic platform for periodontitis, integrating antibacterial, anti-inflammatory, and regenerative functions into a single system. Their tunable structures and responsive behaviors enable precise control over therapeutic release and immune modulation, offering significant advantages over conventional treatments. However, challenges such as long-term biosafety, controlled degradation, and scalable manufacturing must be addressed before clinical translation. Future research should prioritize the design of smart, immunomodulatory MOF systems that synergistically target infection, inflammation, and tissue regeneration in a spatiotemporally controlled manner. With continued innovation, MOF-based therapies hold great potential to revolutionize periodontal care and improve patient outcomes.
